# Yield, NNS and prevalence of screening for DM and hypertension among pulmonary tuberculosis index cases and contacts through single time screening: A contact tracing-based study

**DOI:** 10.1371/journal.pone.0263308

**Published:** 2022-01-28

**Authors:** Shengqiong Guo, Virasakdi Chongsuvivatwong, Min Guo, Shiguang Lei, Jinlan Li, Huijuan Chen, Jiangping Zhang, Wen Wang, Cui Cai

**Affiliations:** 1 Guizhou Center for Disease Control and Prevention, Guiyang, Guizhou, China; 2 Department of Epidemiology, Faculty of Medicine, Prince of Songkla University, Hat Yai, Songkhla, Thailand; 3 Anshun People’s Hospital, Anshun, Guizhou, China; 4 Yunyan Center for Disease Control and Prevention, Guiyang, Guizhou, China; 5 Guiyang Public Health Clinical Center, Guiyang, Guizhou, China; Chiang Mai University, THAILAND

## Abstract

**Introduction:**

Diabetes mellitus (DM), hypertension and pulmonary tuberculosis (PTB) are catastrophic illnesses that collectively lead to increased mortality and premature death. However, the size of the problem and the appropriate approach to deal with the burden is still unclear. We aimed to evaluate the yield, number needed to screen (NNS) to prevent one death or adverse event for screening DM and hypertension and assess the prevalence and contributors to DM and/or hypertension.

**Methods:**

Based on PTB contact tracing, a cross-sectional study was conducted among 801 PTB index cases and 972 household contacts from April 2019 to October 2020 in Guizhou, China. All the participants were screened for DM and hypertension. The yield was calculated as the proportion of newly detected cases among the study subjects, excluding known cases. The NNS was computed by dividing the number needed to treat for risk factors by the prevalence of the unrecognized diseases. The univariate and multivariate logistic regression analyses were applied to determine the independent predictors of DM and/or hypertension.

**Results:**

Of the 1,773 participants, the prevalence of DM and hypertension was 8.7% (70/801) and 15.2% (122/801) in the PTB patients, 3.2% (31/972) and 14.0% (136/972) in the contacts, respectively. The prevalence of DM and/or hypertension was 21.2% (170/801) among the PTB patients and 15.4% (150/972) among their contacts. The screening yields to detect new cases of DM and hypertension among PTB patients were 1.9% and 5.2%, and that in the contacts were 0.8% and 4.8%, respectively. The NNS for DM was 359 for the PTB cases and 977 for the contacts, 299 for PTB cases and 325 for hypertension, respectively. Older age, under or overweight and obesity, family history hypertension and earlier diagnosis of other chronic conditions were the independent predictors for DM and/or hypertension among both PTB cases and their contacts.

**Conclusion:**

Screening for DM and hypertension should be mandated in PTB patients and their household contacts to disclose undetected cases of these two conditions during TB contact tracing, which might reduce the potential cardiovascular disease deaths.

## Introduction

Diabetes mellitus (DM), hypertension and pulmonary tuberculosis (PTB) are still catastrophic illnesses that collectively lead to increased mortality and premature death. These chronic conditions exacerbate the progress of PTB diseases, such as delaying sputum-culture conversion from positive to negative, which has caused an obstacle in the control of PTB [1].

The prevalence of DM and hypertension is consistently found to be higher among PTB patients than among the general population. In India, the prevalence of diabetes was 7.5% for PTB patients and 4.5% for non-PTB patients and that of hypertension was 24.5% and 17.3%, respectively [2]. In South Africa, 26.9% of people with PTB had one and 25.3% had at least two chronic diseases [3]. In a community-based study in China, the prevalence of DM in PTB patients (6.3%) was higher than that in the non-PTB controls (4.7%) with a 3-fold higher odds ratio (OR) [4].

DM and hypertension are usually considered as twin diseases with substantial overlap. They are frequently concurrent, which share a common pathway and risk factors such as obesity, physical inactivity, and unhealthy lifestyle [5]. In addition, some studies reported that substantial undiagnosed cases with the comorbidity of hypertension and DM were observed [6, 7], which indicates some patients are undiagnosed and untreated, probably leading to a lot of complications, even premature deaths. However, little is known about the precise size of the problem and the appropriate approach to deal with the burden is still unclear.

Disease screening is considered to produce yields both in the clinical field and public health. Yield is the measure of previously unrecognized disease, diagnosed as the result of screening and brought to treatment [8]. According to the World Health Organization (WHO), the number needed to screen (NNS) is the number of persons that need to undergo screening in order to diagnose TB among people at-risk on TB [9]. However, a more public health intensive criteria previously proposed by Rembold was the number of people that needed to screen for a given duration to prevent one death or adverse event [10].

In this article, we analyzed and adopted Rembold’s concept of NNS. If the yield is of high value and the NNS is small, the screening is supposed to add value to the resources spent in regular monitoring. Both the yield and NNS of screening for DM and hypertension were rarely evaluated in the settings of PTB control programs. Screening activities for diabetes and hypertension in the general population have increased as health systems continue to evolve [11–13]. That for diabetes and hypertension in PTB patients has also grown recently [6, 14, 15]. It is significant to document the magnitude of yield and NNS of DM and hypertension screening among the two populations.

It is challenging to directly compare PTB patients and the general populations due to the limited actual conditions. Due to the above reasons, we conducted a large-scale PTB household contact tracing to find the magnitude of undetected NCDs. We aimed to 1) evaluate the yield of screening for DM and hypertension; 2) explore the NNS for DM and hypertension to prevent premature death from these two conditions; and 3) assess the prevalence and contributors to DM and/or hypertension. Our results would provide an insight into assessing the strategies for routine screening and control of related diseases for policymakers.

## Materials and methods

### Study design and sample size

This was a cross-sectional study based on a PTB contact-trace program conducted in Guizhou, China from April 1, 2019 to October 30, 2020.

Totally, 116 villages/communities with high PTB incidence in Guizhou were drawn as the study sites. We considered that the chances of having DM or hypertension were more similar among the household members than among the general population. This required adjusting of the sample size with design effect (*Deff*) [16–18], the value of which was assumed as 2.0 in this study. Eventually, the minimum sample size was computed using the infinite population proportion formula with a continuity correction as shown below.

$$N=[Z^{2}{}_{1‐a/2}\;*P\;*\;(1‐P)]\;*\;Deff/d^{2}$$

where p is the prevalence of DM and hypertension of the epidemiology survey with 7.6% and 27.8%, respectively in Guizhou in 2010 [19], *Deff* = 2.0, *d* = 25% × P, and the type I error rate (α) = 0.05.

The formula resulted in a sample size for DM of 1,495 and 409 for hypertension. With the consideration of a 10% rate of non-response, 1661 people were planned to be recruited for DM screening and 454 for hypertension screening.

### Relevant definitions

#### Yield

In different kinds of literature, the yield has different meanings. Firstly, it can be the measure of previously unrecognized disease, diagnosed as the result of screening and brought to treatment. Other forms of yield are provided by persons with known disease who have previously lapsed from treatment [8, 20, 21]. Since we ran this screening to detect the unrecognized cases of DM and hypertension, we confine our interest to the first definition. Our yield was calculated from the number of newly detected diseases among the screened population that excluded those known to be diseased. Note that the yield was calculated in other papers by positive predictive value (PPV) [22, 23]. This was, however, not relevant to the purpose of our study.

#### Number needed to screen (NNS)

The number of people that need to be screened to prevent one death or one adverse event [10]. To calculate the NNS, we added three more terms, the number needed to treat (NNT), absolute risk reduction (ARR) and the prevalence of interested disease (PrC). NNT is the reciprocal of the ARR, defined as the number of people that need to be treated for a given duration, such as five or ten years, to prevent one death or one adverse event [10]. ARR is the number of percentage points the risk goes down if something protective has been done to stop it [24]. The PrC is the prevalence of an interested disease that is unrecognized [10, 25]. Eventually, the NNS is one divided by AAR multiply one divided by PrC.

#### PTB index case

At least two positive results of sputum smear, or positive result of one sputum smear with chest X-ray positive subsequent to two weeks of antibiotic medication, or Xpert MTB/RIF cartridges assay positive [26], or one sputum sample cultured containing bacilli. Household contact: Lived in the same house with an index PTB patient for more than 6 hours per week [27] between 3 months earlier than the diagnosis of the PTB index case and 14 days after the PTB index case initiating anti-tuberculosis treatment.

#### DM

Fasting plasma glucose (FPG) ≥126 mg/dl or random plasma glucose (RPG) ≥200 mg/dl or a previous diagnosis of DM.

#### Prediabetes

FPG at least 110 but below 126 mg/dl [28] according to the parameters set by the American Diabetes Association (2016).

#### Hypertension

Systolic blood pressure (SBP) ≥140 mmHg and/or diastolic blood pressure (DBP) ≥ 90 mmHg or with a history of previously known disease as per WHO criteria. Prehypertension: SBP 130~139 mmHg and/or DBP 85~89 mmHg [29].

#### Salt-intake limit

Over 6 grams/day/adult according to the Dietary Guidelines for Chinese Residents (2016) [30].

#### Oil-intake limit

Over 30 grams/day/adult according to the Dietary Guidelines for Chinese Residents (2016) [30].

#### Smoking

Smoking in the past 12 months, including both daily and non-daily smoking.

#### Drinking

Drinking in the past 12 months, including both daily and non-daily drinking.

### Ethical consideration and guidelines and regulations statement

This protocol was approved by the Ethics Committee of the Faculty of Medicine, Prince of Songkla University, Hat Yai, Thailand (No: 61-335-18-1) and the Ethics Committee of Guizhou Centre for Disease Control and Prevention (No: Q2019-01) before this study was conducted. We confirm that all the methods were carried out in accordance with the relevant human guidelines and regulations.

### Consent to participate

Before the study was conducted, written informed consent was obtained from each study participant. For participants aged under 18 years, the information sheet was sent to their parents or legal guardians. All investigations related to them were initiated with the written informed consent of their parents or legal guardians.

### Study procedure and data collection

Initially, newly diagnosed PTB cases aged 15 years or more and currently on treatment for a duration of 0–6 months and notified to the National Tuberculosis Program from the study site were consecutively retrieved. Pregnant women, mentally disabled persons, and those living alone were excluded from the analysis.

During the patients’ monthly visits to the hospital to obtain their medications, PTB medical staff would approach the patients to obtain informed consent and make an appointment with them for a home visit.

During the home visits, we surveyed up to three contacts aged 15 years or more. The simple random sampling method was used to select respondents when there were more than three adult contacts in any study household.

The presence of DM and hypertension were screened by assessing SBP/DBP and FPG/RPG among all participants following the standard criteria. The participants with different diseases identified from the study were transferred to their local clinics to get appropriate treatment. Those with known diseases who have previously lapsed from treatment were suggested to access their local hospitals to continue medical services.

### Statistical analysis

Data obtained from the questionnaires and medical records reviewing were entered into EpiData version 3.1 (http://www.epidata.dk/). R language and environment version 3.6.3 (https://cran.r-project.org/) was employed for the statistical analysis. Student’s t-test or ANOVA was used to compare age, FPG/RPG and SBP/DBP among groups as appropriate and summarized using the mean and standard deviation. The nonparametric Mann-Whitney U test was performed for continuous variables when data were not normally distributed. Chi-square or Fisher exact tests were applied for categorical variates where appropriate.

The yield was calculated by dividing the number of newly detected diseases by the number of subjects screened, excluding those known to be diseased. The NNS was computed by dividing the NNT for risk factors by PrC. The NNT was computed through one divided by the ARR of the interested disease, which equals the risk off treatment minus the risk on treatment [31]. We obtained the estimates for ARR of DM [31] and hypertension [32] for our study from the previous studies.

The univariate analysis was applied for the risk factors contributing to DM and/or hypertension that were regarded as twin diseases. The considered variables included socio-demographic, behavioral and clinical characteristics, such as gender, age, occupation, monthly income, smoking, drinking, exercising, staying-up-late, meat-eating frequency, and family history of DM and hypertension and other non-communicable diseases (NCDs), presence of other NCDs and the knowledge of edible oil and salt intake limits.

We fitted a multivariate logistic regression model to determine the associated factors. For the outcome variable in the model, we used the subjects who were normal as the referent group and those with DM or hypertension or both of DM & hypertension as the positive group. The independent variables were those having a *P* value < 0.2 in the univariate analysis. The ORs of associated factors to DM and/or hypertension from the final model were demonstrated through two forest plots [33], one for the PTB patients and another for their household contacts.

### Investigation quality control

This study mainly referred to the previous exposure of the research subjects, so recall bias might be caused by the distortion or vague memory of the respondents. We trained the investigators to collect the data with a blind method and investigate skillfully to reduce recall bias. Besides, we addressed missing data via a multiple imputation technique.

## Results

### Sampling and general characteristics

**Fig 1:**
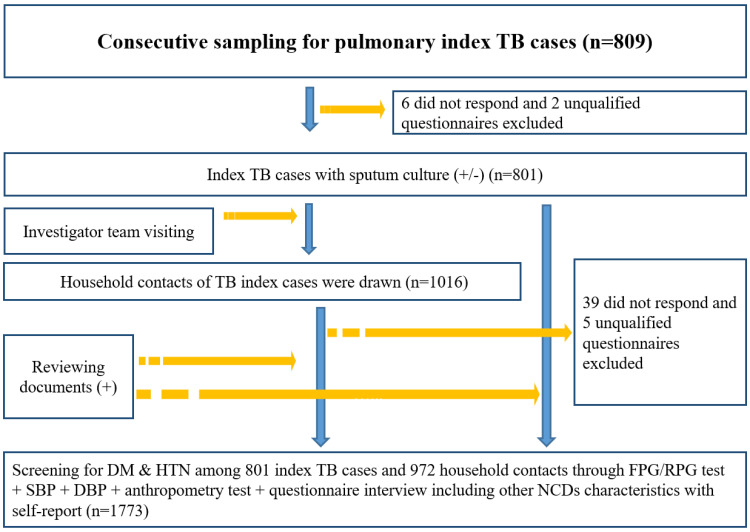
Flowchart of study on yield, NNS and prevalence of DM/hypertension screening, 2020. PTB: tuberculosis. DM: diabetes. HTN: hypertension. NCDs refer to DM, hypertension, dyslipidemia, malignant neoplasm, chronic obstructive pulmonary disease, heart attack and chronic renal disease.

Initially, 809 index PTB cases were recruited, and 1,016 related adult household contacts were selected. Six neither responded nor participated in the disease screening test and were excluded during the in-home visits. Similarly, 39 contacts were excluded as they did not respond and therefore did not participate in the screening process. Later, two PTB index cases and their five household contacts were also excluded due to unqualified information. Eventually, 801 (45.2%) PTB index cases and 972 (54.8%) household contacts were included in the study (Fig 1).

Of the 1,773 participants, aged between 15 and 99 years, the mean (standard deviation, SD) age of PTB patients was 47.6 (19.3) years, with 62.8% males. The family contact group’s mean (SD) age was 46.6 (15.7) years, with 42.8% males. Table 1 compares the background characteristics of the respondents. The PTB cases were significantly older, more likely to be male and had a lower income. The household contacts were more likely to be married or cohabitating than PTB cases. Both groups were primarily peasants and not significantly different in terms of their levels of education attained.

**Table 1:** Socio-demographic characteristics of study participants (n, %).

Demographic Characteristics		Total	Patient	Contact	*P* value
**Total**		1773	801	972	
**Age group (years)**	**15~34**	503(28.4)	254(50.5)	249(49.5)	< 0.001
	**35~59**	814(45.9)	298(36.6)	516(63.4)	
	**60~100**	456(25.7)	249(54.6)	207(45.4)	
**Gender**	**Female**	854 (48.2)	298 (37.2)	556 (57.2)	< 0.001
	**Male**	919 (51.8)	503 (62.8)	416 (42.8)	
**Education**	**Below primary**	833 (47.0)	368 (45.9)	465 (47.8)	0.148
	**Middle school**	784 (44.2)	351 (43.8)	433 (44.5)	
	**University and above**	156 (8.8)	82 (10.2)	74 (7.6)	
**Occupation**	**Clerk**	79 (4.5)	38 (4.7)	41 (4.2)	0.002
	**Student**	90 (5.1)	56 (7)	34 (3.5)	
	**Peasant**	987 (55.7)	418 (52.2)	569 (58.5)	
	**Migrant-laborer**	617 (34.8)	289 (36.1)	328 (33.7)	
**Marriage**	**Single**	105 (5.9)	68 (8.5)	37 (3.8)	< 0.001
	**Married/cohabitating**	1398 (78.8)	559 (69.8)	839 (86.3)	
	**Separated/divorced/widowed**	270 (15.2)	174 (21.7)	96 (9.9)	
**Monthly income (CNY)**	**0~999**	688 (38.8)	365 (45.6)	323 (33.2)	< 0.001
	**1,000~2,999**	660 (37.2)	254 (31.7)	406 (41.8)	
	**3,000~4,999**	302 (17)	124 (15.5)	178 (18.3)	
	**5,000~**	123 (6.9)	58 (7.2)	65 (6.7)	

**Note** CNY: Chinese yuan.

### Prevalence, yield and NNS of DM and hypertension

Table 2 displays the prevalence, the yield and the NNS of screening for DM and hypertension. The prevalence of DM among PTB patients was (56+14)/801 = 8.7%. This was statistically significantly higher than among the contact of (23+8)/972 = 3.2% (*P* value of Chi-square test <0.01). Similarly, the yield or newly detected rate in the PTB group (14/745 = 1.9%) was significantly higher than that in the contact group (8/949 = 0.8%) with *P* value < 0.01. On the contrary, the NNS of the former (359) is lower than that of the latter (977).

**Table 2:** Prevalence, percent yield and NNS of DM/hypertension screening, 2020 (n, %).

**Subject group**	**Non-diabetes (a)**	**Prediabetes (b)**	**Diabetes**		**Yield = [(d)/(a+b+d)]** ×**100 (%)**	**Prevalence (PrC) = [(c+d)/(a+b+c+d)]** ×**100 (%)**	**ARR** ^**#**^	**NNT** ^**##**^	**NNS** ^**###**^
			**Previously known (c)**	**Newly detected (d)**					
**Total**	1,218	454	79	22	1.3	5.7	0.032	31	548
**Patient**	523	208	56	14	1.9*	8.7*	0.032	31	359
**Contact**	695	246	23	8	0.8	3.2	0.032	31	977
**Subject group**	**Non- hypertension (a)**	**Prehypertension (b)**	**Hypertension**		**Yield = [(d)/(a+b+d)]** ×**100 (%)**	**Prevalence (PrC) = [(c+d)/(a+b+c+d)]** ×**100 (%)**	**ARR** ^**#**^	**NNT** ^**##**^	**NNS** ^**###**^
			**Previously known (c)**	**Newly detected (d)**					
**Total**	1,220	295	179	79	5.0	14.6	0.022	45	311
**Patient**	554	125	85	37	5.2	15.2	0.022	45	299
**Contact**	666	170	94	42	4.8	14.0	0.022	45	325

**Note** ^#^ARR: Absolute risk reduction (ARR_DM_ = 0.032, ARR_hypertension_ = 0.022). ^##^NNT: Number needed to treat (NNT = 1/ARR). ^###^NNS: Number needed to screen to save one life (NNS = NNT/PrC). * *P* value <0.05 compared to the contact group.

There was no significant difference in hypertension prevalence between the two groups (15.2% vs. 14.0%). So was the difference in the yield (5.2% vs. 4.8%). Consequently, the NNSs for hypertension of both groups were close (299 vs. 325).

Overall, with many times that the screening yield of hypertension was higher than DM, the NNS for hypertension was consistently lower than that for DM in all groups.

### Breakdown of prevalence of DM and/or hypertension

Table 3 displays the prevalence of DM and/or hypertension and DM comorbid with hypertension. The prevalence of DM and/or hypertension was 21.2% (170/801) among the PTB patients and 15.4% (150/972) among their household contacts. The prevalence of DM comorbid with hypertension was 2.7% (22/801) among the former and 1.7% (17/972) among the latter. High proportions of prediabetes and prehypertension were detected in PTB patients and their families. Except for prediabetes and prehypertension in PTB cases, the prevalence of the other identified diseases of the two groups increased significantly with increasing age.

**Table 3:** Prevalence of DM and/or hypertension and DM & hypertension, 2020 (n, %).

Variable		Total	Gender			Age (year-old)			
			Female	Male	*P* value	15~34	35~59	60~100	*P* value
**Diabetes or hypertension**	**patient**	170(21.2)	56 (18.8)	114 (22.7)	0.23	9 (3.5)	57 (19.1)	104 (41.8)	< 0.001
	**contact**	150(15.4)	83 (14.9)	67 (16.1)	0.68	9 (3.6)	65 (12.6)	76 (36.7)	< 0.001
	***P* value**	0.002							
**Diabetes and Hypertension**	**patient**	22(2.7)	6 (2.0)	16 (3.2)	0.451	0 (0.0)	7 (2.3)	15 (6.0)	< 0.001
	**contact**	17(1.7)	9 (1.6)	8 (1.9)	0.807	1 (0.4)	7 (1.4)	9 (4.3)	0.006
	***P* value**	0.207							
**Prediabetes or prehypertension**	**patient**	302(37.7)	98 (32.9)	204 (40.6)	0.037	75 (29.5)	121 (40.6)	106 (42.6)	0.005
	**contact**	371(38.3)	199 (35.8)	172 (41.3)	0.09	76 (30.5)	207 (40.1)	88 (42.5)	0.013
	***P* value**	0.879							
**Prediabetes and Prehypertension**	**patient**	31(3.9)	8 (2.7)	23 (4.6)	0.25	4 (1.6)	16 (5.4)	11 (4.4)	0.061
	**contact**	45(4.6)	21 (3.8)	24 (5.8)	0.191	4 (1.6)	34 (6.6)	7 (3.4)	0.006
	***P* value**	0.504							

### Univariate analysis for DM and/or hypertension

Taking the presence of DM and/or hypertension as the dependent variable, the univariate analysis was conducted using the Chi-square test or Fisher test. Older age, less education, underweight or obesity, being separated/divorced/widowed, family history of hypertension and other NCDs and an earlier diagnosis of other NCDs were the associated factors of DM and/or hypertension according to the univariate analysis.

Among the PTB patients, DM and/or hypertension was more likely to occur among peasants and those individuals with a family history of DM or with sputum-smear positive results.

In the household contacts, those with a lower monthly income, or knew the limit of salt-intake, or were depressed and cared for their PTB patients were more likely to have DM and/or hypertension (*P* values are available in the S1 Table).

### Forest plots of multivariate logistic regression analysis

With the presence of DM and/or hypertension as the dependent variable, the variables with a *P* value < 0.2 got from the univariate analysis as the independent variables, a multivariate logistic regression model was applied to determine the independent predictors.

**Fig 2:**
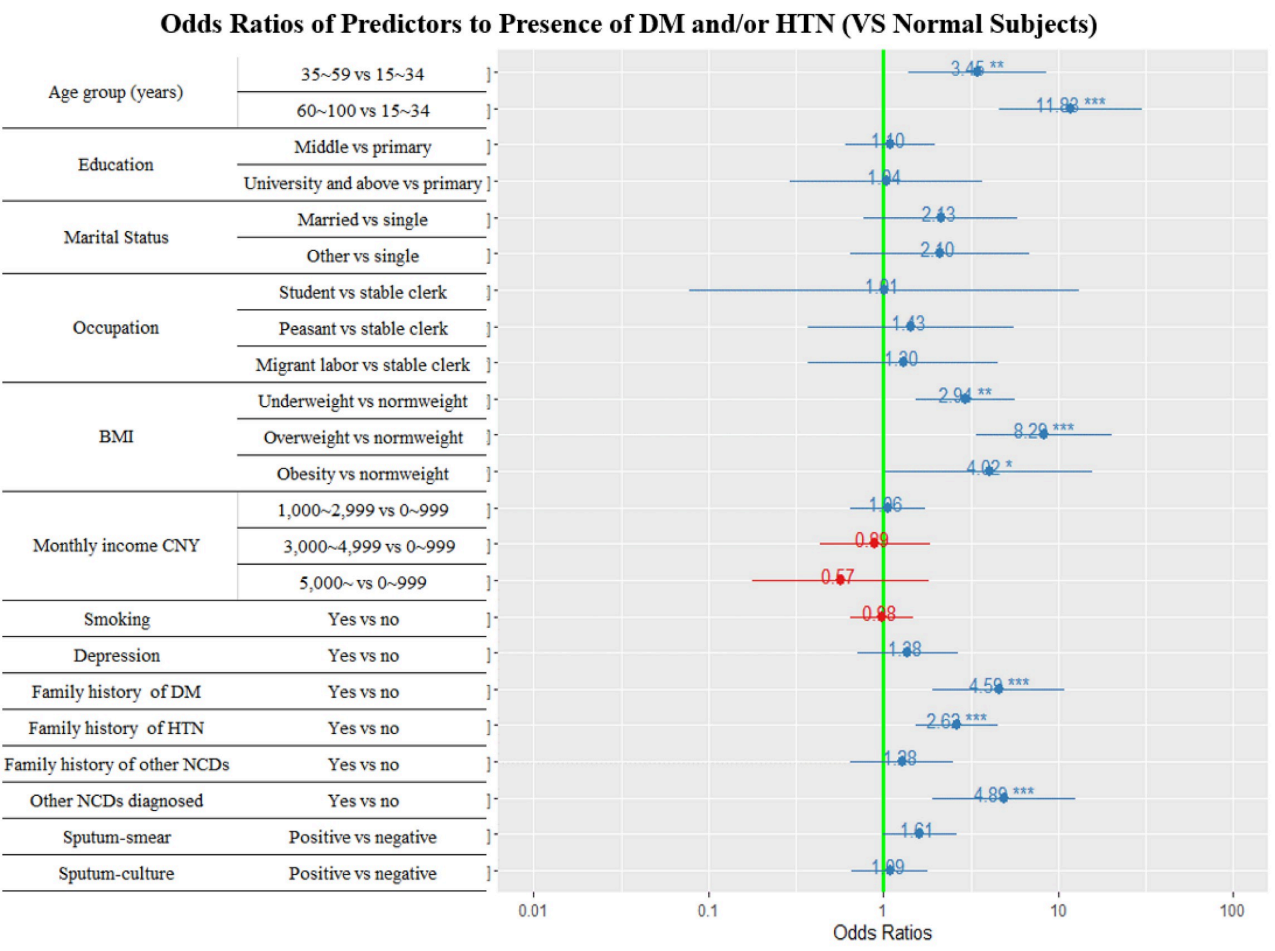
Adjusted odds ratios of association to DM and/or hypertension among the PTB patients, 2020. Blueline refers to 95%CI. * Stands for *P* values, ‘***’ < 0.001 ‘**’ < 0.01 ‘*’ < 0.05. DM: diabetes mellitus. HTN: hypertension. NCDs refer to DM, hypertension, dyslipidemia, malignant neoplasm, chronic obstructive pulmonary disease, heart attack, chronic renal disease. Diagnosed NCDs refer to been diagnosed as NCD patients mentioned above except for DM and hypertension. Other marital statuses: Separated/divorced/widowed.

**Fig 3:**
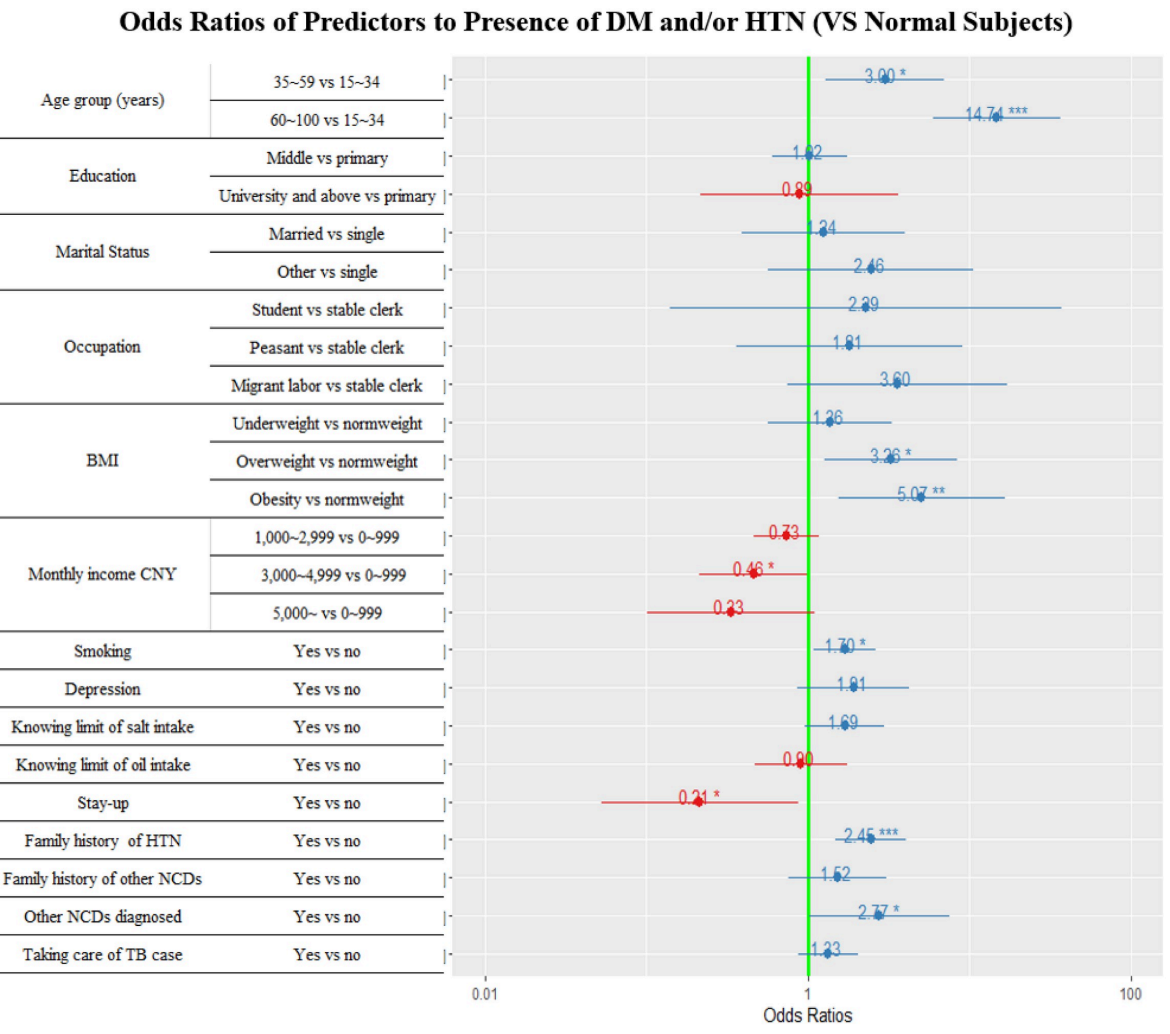
Adjusted odds ratios of association to DM and/or hypertension among the household contacts, 2020. Blueline refers to 95%CI. * Stands for *P* values, ‘***’ < 0.001 ‘**’ < 0.01 ‘*’ < 0.05. DM: diabetes mellitus. HTN: hypertension. NCD refers to DM, hypertension, dyslipidemia, malignant neoplasm, chronic obstructive pulmonary disease, heart attack, chronic renal disease. Diagnosed NCD refers to been diagnosed as NCD patient mentioned above except for DM and hypertension. Other marital statuses: Separated/divorced/widowed.

Figs 2 and 3 display the forest plots of the independent predictors of DM and/or hypertension among PTB index cases and their household contacts. Older age, under or overweight and obesity, hypertension family history, and earlier diagnosis of other NCDs were the collectively independent predictors of DM and/or hypertension for both PTB index cases and their household contacts. Having a family history of DM was the positively associated factor of DM and/or hypertension among the PTB cases (Fig 2). Lower-income and smoking were significantly associated with DM and/or hypertension in the household contacts (Fig 3).

## Discussion

The prevalence of DM was more than two times higher among the index PTB cases than their household contacts, whereas the prevalence of hypertension in the two groups was close. Undetected hypertension was more common than undetected DM in both groups. As a result, the yield of screening for hypertension is higher and the NNS was lower than that for DM.

Our prevalence of DM or hypertension is slightly lower than in a survey conducted in the general population in 2010 of Guizhou (7.6% and 27.8%, respectively) [19]. The lower prevalence might be because the participants in our study were younger than those in the survey in 2010 (≥15 years vs. ≥18 years). In the current study, DM and hypertension co-existed in 2.2% (2.7% among the PTB cases and 1.7% in the contacts), slightly lower than 4.5% in the general population of an Indian study [34], while the DM prevalence among PTB cases was higher than 6.0% reported in a study from Angola [6]. PTB patients comorbid with DM have a lower concentration of anti-tuberculosis drugs and a higher risk of drug toxicity than tuberculosis patients without DM [35], indicating that PTB patients should be remained a priority group for medical services on chronic conditions. However, chronic diseases in their family contacts also should be properly managed because we observed a significant proportion of prediabetes and prehypertension in these participants who were considered a substitute for the general population. Furthermore, the household contacts might carry similar susceptible genes with their index cases, such as speckle 110 (SP110), or human leukocyte antigen (HLA), which might be the possibly susceptible genes to the occurrence of DM or TB disease [36–38].

Just like PTB, early detection of DM and hypertension is of value in preventing related complications. In our study, the NNS for detecting DM was 359 in PTB patients and 977 in the household contacts. The respective numbers for hypertension were 299 and 325. The values of NNS in PTB cases were lower than that in their family in both DM and hypertension; especially for DM, the difference even reached three times. The NNS indicates how many persons needed to screen for a given duration to prevent one death or adverse event. The lower the NNS is, the more pivotal the screening would be. Therefore, in our sampled participants, 359 PTB index cases, or 977 household contacts, are needed to be screened for DM to prevent one death or adverse event for a given duration if the detection was followed by a routine treatment for the patients [10]. The number of contacts is 3-fold over that of PTB cases, which might be because the risk of developing DM is higher in the PTB group than in their household contacts. In other words, the TB patients’ screening of DM and hypertension should not be missed, and their contacts must be investigated during the contact tracing activities. Our NNS number of hypertension (311) is similar to 274 in a study of Rembold [10]. Note that the NNS numbers were lower for hypertension than that for DM as hypertension is known to have an immense contribution to cardiovascular disease death in the population.

The NNS matters and so does the yield. The yield reflects the importance of these sample screenings among the PTB cases and their families. The greater the yield is, the more probability of the interesting disease the screened people would have. The estimated percent yield in the PTB cases was almost three times (1.9%) over their family (0.8%) for DM addition to the known DM cases, which had further addressed the more priority of medical service related to DM the PTB cases should have [10]. The yield values of screening hypertension in the PTB cases and household contacts were similar. However, our data point out that neglected or undetected hypertension is more common. The yield of hypertension was 3 to 6 times higher than that of DM. More importantly, blood pressure screening can save more lives than DM screening. Thus, hypertension screening should always accompany DM screening, and chronic conditions should be investigated as routine screening programs. This approach is particularly justified in some countries with obtaining the high yield of newly detected cases [11–13].

In our study, older age, under or overweight and obesity, family history of hypertension and earlier diagnoses of other NCDs were identified as the contributors to DM and/or hypertension. Disease screening among critical populations is suggested regarding these risk factors since it may bring more benefits [39–41].

Our study is in line with the previous studies on yield and NNS for screening DM and hypertension in PTB patients and their families [11–14]. The values of NNS and yield were not directly comparable due to the difference in definitions of the two terms. Yet, our study posts an insight into the chronic conditions among households with a PTB case, which makes significant implications in the control programs for TB and NCDs.

### Limitations

There were some limitations in this study. First, it is challenging to address which appeared first between PTB and DM and/or hypertension due to the cross-sectional design and the nature of the three illnesses. Second, the subjects included only index PTB patients and their household contacts. Although household contacts are considered a substitute for the general population, the two populations might have different socio-demographic characteristics. Therefore, the study was unable to directly compare the prevalence data of DM and hypertension, prediabetes and prehypertension between PTB cases and the general population. Prudence should be observed when the results of this study are generalized.

## Conclusion

Screening for DM and hypertension should be mandated in PTB patients and their household contacts to disclose undetected cases of these two conditions during the TB contact tracing, which might reduce the potential cardiovascular disease deaths.

## Supporting information

S1 Checklist STROBE statement—checklist of items that should be included in reports of observational studies. (DOCX)

S1 Table Univariate analysis for DM and/or hypertension. (PDF)

S1 File Study protocol & questionnaire for study on yield, NNS and prevalence of screening for diabetes mellitus and hypertension. (PDF)

S1 Data Original data for study on yield, NNS and prevalence of screening for diabetes mellitus and hypertension. (XLSX)
