# Supplementary material for: Yield, NNS and prevalence of screening for DM and hypertension among pulmonary tuberculosis index cases and contacts through single time screening: A contact tracing-based study
Source: PLoS One. 2022 Jan 28;17(1):e0263308. doi: 10.1371/journal.pone.0263308 (PMC8797235; doi:10.1371/journal.pone.0263308)
Supplement: S1 Checklist — (DOCX) [file pone.0263308.s001.docx]

..STROBE Statement—checklist of items that should be included in reports of observational studies

|  | Item No. | Recommendation | Page  No. | Relevant text from manuscript |
| --- | --- | --- | --- | --- |
| **Title and abstract** | 1 | (*a*) Indicate the study’s design with a commonly used term in the title or the abstract | P2 | A tuberculosis contact trace-based cross-sectional study was conducted… |
|  |  | (*b*) Provide in the abstract an informative and balanced summary of what was done and what was found | P3 | Screening for diabetes and hypertension as well as related risk factors in the existing tuberculosis program produces high additional yield and it is feasible and desperately needed in the era with COVID-19 pandemic. |
| Introduction | | | |  |
| Background/rationale | 2 | Explain the scientific background and rationale for the investigation being reported | P5-6 | However, there is a paucity of research issues on the fundamental numbers of the AY and the NNS to find a new case for DM or HTN among the households with a tuberculosis patient as well as the related risk factors as well. It is difficult to… |
| Objectives | 3 | State specific objectives, including any pre-specified hypotheses | P5 | Therefore, we hypothesized that TB index cases had a higher prevalence of DM and HTN than… |
| Methods | | | |  |
| Study design | 4 | Present key elements of study design early in the paper | P6 | 2.1 Study Design: A cross-sectional study based on TB contact-trace was conducted in Guizhou, China from April 1, 2019 to October 30, 2020. Eleven out of 88 counties/districts were randomly selected as the study sites  2.2 Relevant Definitions DM&HTN… |
| Setting | 5 | Describe the setting, locations, and relevant dates, including periods of recruitment, exposure, follow-up, and data collection | P6 | 2.1 Study Design: A cross-sectional study based on TB contact-trace was conducted in Guizhou, China from April 1, 2019 to October 30, 2020. Eleven out of 88 counties/districts were randomly selected as the study sites; Initially, newly diagnosed index cases of TB notified to the National Tuberculosis Program from the study site under the age of 15 years and currently on treatment for 0-6 months were consecutively retrieved. During a patient's monthly visit to the hospital for medication, the TB medical staff would contact the patient through informed consent and a list of respondents, obtain informed consent, conduct an investigation, and then make an appointment with the patient for a home visit… |
| Participants | 6 | (*a*) *Cohort study*—Give the eligibility criteria, and the sources and methods of selection of participants. Describe methods of follow-up  *Case-control study*—Give the eligibility criteria, and the sources and methods of case ascertainment and control selection. Give the rationale for the choice of cases and controls  *Cross-sectional study*—Give the eligibility criteria, and the sources and methods of selection of participants | P8 | 2.6 Study Procedure and Data Collections: …under the age of 15 years and currently on treatment for 0-6 months were consecutively retrieved. Those on a TB retreatment program, pregnant women, the mentally retarded, or those living alone were exclude. …Household contact: Lived in the same house as an index TB patient for more than 6 hours per week from 3 months before diagnosis and 14 days after the initiation of anti-tuberculosis treatment diagnosed as TB followed the procedures. |
|  |  | (*b*) *Cohort study*—For matched studies, give matching criteria and number of exposed and unexposed  *Case-control study*—For matched studies, give matching criteria and the number of controls per case |  |  |
| Variables | 7 | Clearly define all outcomes, exposures, predictors, potential confounders, and effect modifiers. Give diagnostic criteria, if applicable | P6-7,10 | The AY of DM from screening was calculated as the number of newly diagnosed DM / (previously known + newly diagnosed DM) × 100%. The NNS to find a new case of DM was calculated as the reciprocal of the prevalence of newly diagnosed DM after excluding self-reported previously known cases of DM. The same algorithm was applied to hypertension. The considered correlates included socio-demographic, behavioral and clinical characteristics, such as gender, age, ethnicity, occupation, monthly income, smoking, drinking alcohol, doing exercise, stay-up-late, meat taking frequency, and family history of DM and HTN and other NCDs, a subject diagnosed as other NCDs and the knowledge of edible oil and salt intake limits…  Index TB case: At least two positive results of sputum smear, or positive result of one sputum smear with chest X-ray positive subsequent to two weeks of antibiotic medication, or cartridge-based tests such as Gene X-pert® (X-pert) positive, or one sputum sample cultured containing bacilli…  DM: Fasting plasma glucose (FPG) ≥126 mg/dl or random plasma glucose (RPG) ≥200 mg/dl or a previous diagnosis of DM. Prediabetes refers to FPG at least 110 but below 126 mg/dl according to the parameters set by the American Diabetes Association (2016).  HTN: Systolic blood pressure (SBP) ≥140 mmHg and/or DBP ≥90 mmHg or with a history of previously known disease as per WHO criteria. Prehypertension: Diagnosed by SBP as 130~139 mmHg and/or DBP as 85~89 mmHg. |
| Data sources/ measurement | 8* | For each variable of interest, give sources of data and details of methods of assessment (measurement). Describe comparability of assessment methods if there is more than one group | P8,10 | 2.6 Study Procedure and Data Collections  Initially, newly diagnosed index cases of TB notified to the National Tuberculosis Program from the study site…  The ORs of the results from the multinomial logistic regression were demonstrated through the two forest plots, one for the DM/HTN of tuberculosis patients and the other for their household contacts…  The AY of DM from screening was calculated as the number of newly diagnosed DM / (previously known + newly diagnosed DM) × 100%. The NNS to find a new case of DM was calculated as the reciprocal of the prevalence of newly diagnosed DM after excluding self-reported previously known cases of DM. The same algorithm was applied to hypertension. |
| Bias | 9 | Describe any efforts to address potential sources of bias | P10,11 | 2.9 Investigation quality control: It mainly refers to the previous exposure of the research subjects, so recall bias might be caused by the distortion or incomplete memory of the respondents. Training the investigators taking the way of blind data collection and emphasis on questionnaire questioning methods and survey techniques to reduce the recall bias. |
| Study size | 10 | Explain how the study size was arrived at | P9 | N = [Z^2^_1-a/2_ p(1-p) * *Deff*] / *d*^2^ |

Continued on next page

| Quantitative variables | 11 | Explain how quantitative variables were handled in the analyses. If applicable, describe which groupings were chosen and why | P9, 5 | For quantitative variables, such as age and monthly income, they were group a continuous exposure variable to create new categorical variables, which have important consequences for the later analyses.  However, there is a paucity of research issues on the fundamental numbers of the AY and the NNS to find a new case for DM or HTN among the households with a tuberculosis patient as well as the related risk factors as well. It is difficult to compare the DM or HTN risk among well-established TB cases detected within a health setting and the general population. While it might be possible to obtain a similar prevalence among household contacts instead of the general population through household contact tracing for TB. Therefore, we hypothesized that TB index cases had a higher prevalence of DM and HTN than that of household contacts in the case of the presence of positive interaction between TB and DM or HTN to conduct a household-based contact-tracing study. |
| --- | --- | --- | --- | --- |
| Statistical methods | 12 | (*a*) Describe all statistical methods, including those used to control for confounding;  (*b*) Describe any methods used to examine subgroups and interactions  (*c*) Explain how missing data were addressed  (*d*) *Cross-sectional study*—If applicable, describe analytical methods taking account of sampling strategy  (*e*) Describe any sensitivity analyses | P9-11 | Line 161-196 all the part of 2.8 Statistical Analysis and 2.9 Investigation quality control: Data obtained from the questionnaires and medical record review were entered into EpiData version 3.1 (http://www.epidata.dk/) and R version 3.6.3 (https://cran.r-project.org/) was employed for the statistical analysis with “epicalc” and other packages. Descriptive statistics was used for general characteristics of the participants, reporting frequencies, proportions, means, medians, and ranges where appropriate. For quantitative variables, such as age and monthly income, they were group a continuous exposure variable to create new categorical variables, which have important consequences for the later analyses. Student’s t-test or ANOVA test was employed for age, FPG/RPG and SBP/DBP comparison between/among groups as appropriate, which were summarized using the mean and standard deviation. A nonparametric Mann-Whitney U test was performed for continuous variables analysis when data are not normally distributed. Chi-square and Fisher exact tests were applied for categorical variates where appropriate. The AY of DM from screening was calculated as the number of newly diagnosed DM / (previously known + newly diagnosed DM) × 100%. The NNS to find a new case of DM was calculated as the reciprocal of the prevalence of newly diagnosed DM after excluding self-reported previously known cases of DM. The same algorithm was applied to hypertension. The univariate analysis was applied for the associated factors relating to DM/HTN.  The considered correlates included socio-demographic, behavioral and clinical characteristics, such as gender, age, ethnicity, occupation, monthly income, smoking, drinking alcohol, doing exercise, stay-up-late, meat taking frequency, and family history of DM and HTN and other NCDs, a subject diagnosed as other NCDs and the knowledge of edible oil and salt intake limits.  Later, taking DM/HTN as the dependent variable, other positive correlates as the independent variables, we created the fittest multinomial logistic regression model with the lowest Akaike’s information criterion (AIC) for the multivariable correlates analysis.  The ORs of the results from the multinomial logistic regression were demonstrated through the two forest plots, one for the DM/HTN of tuberculosis patients and the other for their household contacts.  For the addressing of missing data, deleting the cases with missing values, or prudently select possible value interpolation to fill the missing values, such as mean interpolation, the use of homogeneous mean interpolation, maximum likelihood estimation or multiple interpolation. |
| Results | | | | |
| Participants | 13* | (a) Report numbers of individuals at each stage of study—eg numbers potentially eligible, examined for eligibility, confirmed eligible, included in the study, completing follow-up, and analysed | P6 | Index TB case: At least two positive results of sputum smear, or positive result of one sputum smear with chest X-ray positive subsequent to two weeks of antibiotic medication, or cartridge-based tests such as Gene X-pert® (X-pert) positive, or one sputum sample cultured containing bacilli.  Household contact: Lived in the same house as an index TB patient for more than 6 hours per week from 3 months before diagnosis and 14 days after the initiation of anti-tuberculosis treatment diagnosed as TB followed the procedures. |
|  |  | (b) Give reasons for non-participation at each stage | P11 | Fig. 2 |
|  |  | (c) Consider use of a flow diagram | P11 | Fig. 2 |
| Descriptive data | 14* | (a) Give characteristics of study participants (eg demographic, clinical, social) and information on exposures and potential confounders | P12 | Table 1 |
|  |  | (b) Indicate number of participants with missing data for each variable of interest |  |  |
|  |  | (c) *Cohort study*—Summarise follow-up time (eg, average and total amount) |  |  |
| Outcome data | 15* | *Cohort study*—Report numbers of outcome events or summary measures over time |  |  |
|  |  | *Case-control study—*Report numbers in each exposure category, or summary measures of exposure |  |  |
|  |  | *Cross-sectional study—*Report numbers of outcome events or summary measures | P13,14 | Table 2, 3 |
| Main results | 16 | (*a*) Give unadjusted estimates and, if applicable, confounder-adjusted estimates and their precision (eg, 95% confidence interval). Make clear which confounders were adjusted for and why they were included  (*b*) Report category boundaries when continuous variables were categorized  (*c*) If relevant, consider translating estimates of relative risk into absolute risk for a meaningful time period | P16 | Fig 3, Fig4 |

Continued on next page

| Other analyses | 17 | Report other analyses done—eg analyses of subgroups and interactions, and sensitivity analyses | P15 | For TB patients, DM/HTN was more likely to appear among peasant, population, those respondents with DM (48.9% vs 19.6%) and sputum smear-positive (28.0% vs 17.0%) exclusively. Among household contacts, those who with lower monthly income, smoke, alcohol using disorder, salt excessive intake (19.3% vs 13.9%), depression (31.4% vs 14.5%) and caring of TB patients (20.3% vs 11.0%) were more likely to suffer from DM/HTN (Supplementary Table S1). | |
| --- | --- | --- | --- | --- | --- |
| Discussion | | | | | |
| Key results | 18 | Summarise key results with reference to study objectives | P16 | | Most of the subjects were peasants and Han ethnicity with a low level of education as well as low income. The TB index cases were different from the household contacts in terms of age, gender, occupation, and marital status. Both the prevalence of DM/HTN and DM comorbid with HTN was higher among the TB patients compared to that among their household contacts, increasing with age significantly. DM and HTN screening in these subjects gave an AY of 21.8% to detect DM cases, and that of 30.6% to detect HTN. The NNS to find a new DM or HTN case was 77 and 20, respectively. Older age, under or overweight, presence of HTN family history, subjects diagnosed as NCD earlier were the independent correlates to DM/HTN for both TB index cases and their household contacts. |
| Limitations | 19 | Discuss limitations of the study, taking into account sources of potential bias or imprecision. Discuss both direction and magnitude of any potential bias | P19 | | Limitations: There were limitations in this study. First, for the patients, it is hard to address which appeared first between TB and DM/HTN due to both the design of cross-sectional and the natures of the three chronic diseases. Second, the subjects included only index TB patients and their household contacts, no general population was included, so the data did not allow direct comparison of DM and HTN prevalence, PDM and PHTN between TB cases and the general population. |
| Interpretation | 20 | Give a cautious overall interpretation of results considering objectives, limitations, multiplicity of analyses, results from similar studies, and other relevant evidence | P16-19 | | L281-340, all the part of 4. Discussion. |
| Generalisability | 21 | Discuss the generalisability (external validity) of the study results | P19 | | Second, the subjects included only index TB patients and their household contacts, no general population was included, so the data did not allow direct comparison of DM and HTN prevalence, PDM and PHTN between TB cases and the general population. It should be cautious when the results of this study are generalized. |
| Other information | |  | | | |
| Funding | 22 | Give the source of funding and the role of the funders for the present study and, if applicable, for the original study on which the present article is based | P20 | | Funding Sources: 1. The Guizhou Science and Technology project (S.G., grant number [2020]1Y355); 2. The Post-subsidy Fund Project of National Natural Science Foundation of China in 2019: Special project for the cultivation of novel academic seedlings and innovative exploration of Guizhou Provincial Center for Disease Control and Prevention 2019 (S.L., grant number 2019); 3. Thailand's Education Hub for ASEAN Countries (TEH-AC) Scholarship (S.G., grant number TEH-AC 054/2017); 4. The research reported in this publication was partially supported by the Fogarty International Center and the National Institute of Allergy and Infectious Diseases, of the National Institutes of Health (V.C., grant number D43 TW009522). The content is solely the responsibility of the authors and does not necessarily represent the official views of the funding agencies. |

*Give information separately for cases and controls in case-control studies and, if applicable, for exposed and unexposed groups in cohort and cross-sectional studies.

**Note:** An Explanation and Elaboration article discusses each checklist item and gives methodological background and published examples of transparent reporting. The STROBE checklist is best used in conjunction with this article (freely available on the Web sites of PLoS Medicine at http://www.plosmedicine.org/, Annals of Internal Medicine at http://www.annals.org/, and Epidemiology at http://www.epidem.com/). Information on the STROBE Initiative is available at www.strobe-statement.org.
