# Supplementary material for: Yield, NNS and prevalence of screening for DM and hypertension among pulmonary tuberculosis index cases and contacts through single time screening: A contact tracing-based study
Source: PLoS One. 2022 Jan 28;17(1):e0263308. doi: 10.1371/journal.pone.0263308 (PMC8797235; doi:10.1371/journal.pone.0263308)
Supplement: S1 Table — (PDF) [file pone.0263308.s002.pdf]

S1 Table. Univariate analysis for independent predictiors of DM and/or hypertension, 2020 [vs. Non-DM and non-hypertension (n, %)]

Variables		Patient	*P* value	Contact	*P* value
**Total**		**170**		**150**	
**Gender**	**Female**	56 (18.8)	0.228	83 (14.9)	0.679
	**Male**	114 (22.7)		67 (16.1)	
**Age (year-old)**	**15~**	9 (3.5)	< 0.001	9 (3.6)	< 0.001
	**35~**	57 (19.1)		65 (12.6)	
	**60~**	104 (41.8)		76 (36.7)	
**Ethnicity**	**Han**	135 (23.6)	0.022	120 (16.7)	0.276
	**Buyi**	15 (14.0)		12 (12.8)	
	**Miao**	8 (11.3)		10 (9.9)	
	**Other**	12 (24.0)		8 (13.8)	
**Education**	**Primary and below**	104 (28.3)	< 0.001	87 (18.7)	0.005
	**Middle school**	56 (16.0)		59 (13.6)	
	**University and above**	10 (12.2)		4 (5.4)	
**Religion**	**No**	152 (21.1)	0.746	131 (15.4)	0.739
	**Buddhism**	7 (24.1)		9 (16.1)	
	**Christianism**	0 (0.0)		0 (0.0)	
	**Taoism**	0 (0.0)		0 (0.0)	
	**Other**	11 (25.0)		10 (18.5)	
**Marital Status**	**Single**	7 (4.0)	< 0.001	5 (5.2)	0.003
	**Married/cohabitating**	142 (25.4)		136 (16.2)	
	**Separated/divorced/widowed**	21 (30.9)		9 (24.3)	
**Occupation**	**Clerk**	7 (18.4)	0.002	4 (9.8)	0.065
	**Student**	1 (1.8)		1 (2.9)	
	**Peasant**	99 (23.7)		85 (14.9)	
	**Migrant labor**	63 (21.8)		60 (18.3)	
**Monthly income (CNY)**	**0~**	78 (21.4)	0.882	68 (21.1)	< 0.001
	**1000~**	56 (22.0)		61 (15.0)	
	**3000~**	26 (21.0)		16 (9.0)	
	**5000~**	10 (17.2)		5 (7.7)	
**BMI**	**Norm-weight**	15 (8.6)	< 0.001	8 (9.4)	< 0.001
	**Underweight**	123 (22.2)		83 (12.5)	
	**Overweight**	26 (48.1)		44 (24.2)	
	**Obesity**	6 (33.3)		15 (38.5)	
**Smoke**	**No**	80 (18.3)	0.037	97 (14.0)	0.070
	**Yes**	90 (24.7)		53 (18.9)	
**Alcohol consumption**	**No**	103 (20.3)	0.545	105 (15.2)	0.872
	**Yes**	67 (22.9)		45 (15.9)	
**Know salt-intake limitation**	**No**	124 (20.5)	0.407	98 (13.9)	0.047
	**Yes**	46 (23.6)		52 (19.3)	
**Know oil-intake limitation**	**No**	142 (21.8)	0.433	117 (14.6)	0.143
	**Yes**	28 (18.5)		33 (19.4)	
**Regularly serve of fruit-vegetable**	**No**	138 (21.3)	0.968	36 (18.7)	0.203
	**Yes**	32 (20.8)		114 (14.6)	
**Regularly serve of meat**	**No**	42 (22.6)	0.679	43 (19.7)	0.059
	**Yes**	128 (20.8)		107 (14.2)	
**Physical exercise**	**No**	164 (21.2)	1.000	147 (15.4)	1.000
	**Yes**	6 (22.2)		3 (15.8)	
**Depression**	**No**	146 (20.4)	0.126	134 (14.5)	0.002
	**Yes**	24 (28.2)		16 (31.4)	
**Staying up late**	**No**	153 (21.4)	0.880	147 (16.0)	0.061
	**Yes**	17 (20.0)		3 (5.6)	
**Family history of DM**	**No**	148 (19.6)	< 0.001	139 (15.1)	0.329

	**Yes**	22 (48.9)		11 (21.2)	
**Family history of HTN**	**No**	115 (17.2)	< 0.001	103 (12.4)	< 0.001
	**Yes**	55 (41.0)		47 (33.1)	
**Family history of other NCDs except for DM and HTN**	**No**	141 (19.5)	< 0.001	121 (13.6)	< 0.001
	**Yes**	29 (36.7)		29 (34.1)	
**Other NCDs diagnosed except for DM and HTN**	**No**	145 (18.9)	< 0.001	133 (14.1)	< 0.001
	**Yes**	25 (73.5)		17 (56.7)	
**Sputum smear**	**Negative**	87 (17.2)	< 0.001	/	/
	**Positive**	83 (28.0)		/	
**Sputum culture**	**Negative**	110 (19.7)	0.136	/	/
	**Positive**	60 (24.7)		/	
**Share room with PTB case**	**No**	/	/	96 (14.5)	0.281
	**Yes**	/		54 (17.4)	
**Care of PTB case**	**No**	/	/	56 (11.0)	< 0.001
	**Yes**	/		94 (20.3)	

Note: DM: diabetes mellitus; HTN: hypertension; NCDs refers to DM, HTN, dyslipidemia, malignant neoplasm, chronic obstructive pulmonary disease, heart attack, chronic renal disease here only; Diagnosed NCD, or NCD family history, here NCD refers to been diagnosed as NCD patient mentioned above except for DM and HTN here only; Other Status of marital status: Cohatibrating/Separated/Divorced/Widowed; Oil-intake limit refers to intake the edible oil over 30 grams/day/adult according to the Dietary Guidelines for Chinese Residents (2016). Salt-intake limit refers to intake edible salt over 6 grams/day/adult according to the Dietary Guidelines for Chinese Residents (2016). Smoke refers to smoking in the past 12 months, including both daily and non-daily smoking. Alcohol drinking refers to drinking in the past 12 months, including both daily and non-daily drinking. Regularly serve of meat/fruit/vegitable indicates the frequency number of taking the item is 3 times or greater per week. "/" means there is a lack of this item in the corresponding variable.
