# Supplementary material for: Yield, NNS and prevalence of screening for DM and hypertension among pulmonary tuberculosis index cases and contacts through single time screening: A contact tracing-based study
Source: PLoS One. 2022 Jan 28;17(1):e0263308. doi: 10.1371/journal.pone.0263308 (PMC8797235; doi:10.1371/journal.pone.0263308)
Supplement: S1 File — (PDF) [file pone.0263308.s003.pdf]

Study protocol of yield, NNS and prevalence of screening for Diabetes and hypertension

1. Title of the study

Yield, NNS and prevalence of screening for DM/hypertension among tuberculosis index cases and contacts through single time screening: a contact tracing-based study.

2. Introduction

Recent data for the global burden of disease presented a large number of countries are confronted a huge burden of disease from both chronic non-communicable diseases (NCDs) and communicable diseases (CDs) [1]. NCDs and their risk factors play an important role at the population level in CDs transmissions, such as pulmonary tuberculosis (PTB) epidemics. The prevalence of NCDs, such as diabetes (DM), hypertension (HTN) increase at relatively high speed with the reasons of an aging population, rapid urbanization, changes in environmental factors and lifestyle changes[2].

The prevalence of DM and hypertension is consistently found to be higher among TB patients than among the general population. In India, the prevalence of diabetes was 7.5% for PTB patients and 4.5% for non-TB patients and that of hypertension was 24.5% and 17.3%, respectively[3]. In South Africa, 26.9% of people with TB had one and 25.3% had at least two chronic diseases[4]. In a study in China, the prevalence of DM in TB patients (6.3%) was 3-time higher than that in the non-TB controls (4.7%)[5].

DM and hypertension are usually considered as twin diseases with substantial overlap. They are frequently concurrent, which share a common pathway and risk factors such as obesity, physical inactivity, and unhealthy lifestyle[6]. A high comorbid diseases pattern was found for PTB patients, with 37% HTN and 12% DM according to a primary care study in South Africa based on the analysis of treatment prescriptions[7]. A similar finding was reported in a study of N Folb *et al* that considerable multi-morbidity was found and unmet treatment needs existing with 4% PTB prevalence in HTN group received no treatment or medicines, 6.5% in the DM group and 49.0% in the respiratory diseases group among NCDs patients[8]. In addition, some studies reported that substantial undiagnosed cases with the comorbidity of hypertension and DM were observed both in the general population and among PTB patients[9,10], which

indicates some NCDs patients are undiagnosed and untreated, probably leading to a lot of complications, even premature deaths. However, little is known about the precise size of the problem and the appropriate approach to deal with the burden is still unclear.

Disease screening is considered to produce yield both in the clinical field and public health. Yield is the measure of previously unrecognized disease, diagnosed as the result of screening and brought to treatment [11]. At the same time, clarifying how many people should be detected to save one life is important. The number needed to screen (NNS) is the number of people that needed to screen for a given duration to prevent one death or adverse event[12]. If the yield is of high value and the NNS is small, the screening is supposed to be relatively cost-effective. Both the yield and NNS of screening for DM and hypertension have rarely been evaluated in the settings of PTB control programs. Recently, screening for DM and hypertension in PTB patients is increasing[9,13,14]. It is important to clarify the magnitude of the yield and the NNS for screening DM and hypertension among the community people and PTB population. However, it is difficult to directly compare the problems between the PTB patients and the general population due to the limited actual conditions. In terms of the current well-established household contact tracing program for PTB patients, we assumed that the yield and NNS of screening in contacts of PTB cases are similar to that in the general population. Therefore, We will conduct a large-scale study based on household contact-tracing among TB households with a TB case.

3. Objective(s) of the study

To evaluate the yield, number needed to screen to prevent one death or adverse event (NNS) for screening DM and hypertension and assess the prevalence and contributors to DM and/or hypertension.

4. Research methodology

4.1 Study design

This is a cross-sectional study based on a PTB contact-trace program conducted from April 1, 2019 to October 30, 2020 in Guizhou, China, which is located in Guizhou province and stands the top 4 highest burdens of tuberculosis with high burden of NCDs in China.

4.2 Target population

Tuberculosis patients and their household contacts.

4.3 Research Subjects

4.3.1 Inclusion criteria

PTB index cases: Newly diagnosed TB cases aged 15 years or more and currently on treatment for a duration of 0-6 months and notified to the National Tuberculosis Program from the study site were consecutively retrieved.

Household contact: Aged 15 years or more, lived in the same house with an index TB patient for more than 6 hours per week [15] between 3 months earlier than the diagnosis of the TB index case and 14 days after the TB index case initiating anti-tuberculosis treatment.

4.3.2 Exclusion criteria

Pregnant women, mentally disabled persons, and those living alone were excluded from the analysis.

4.3.3 Discontinuation criteria

All the subjects are free to withdraw off this study if they do not to continue anymore. In addition, if they are not suitable to continue, such as other sudden illness or events or accidents occurring, they are free to withdraw off this study. It is unlikely to terminate unless there is a natural disaster or any other special events.

4.4 Sample size calculation

This is a cross-sectional study based on a TB contact-trace study, which will be conducted in Guizhou, China from April 1, 2019 to October 30, 2020. Totally, 116 villages/communities with high PTB incidence in Guizhou will be drawn as the study sites. We consider that the chances of having DM or HTN were more similar among household members than the general population. This requires adjusting of the sample size with design effect (deff) [16–18], the value of which is assumed as 2.0 in this study. Eventually, the minimum sample size would be computed using the infinite population proportion formula with a continuity correction as shown below.

$$N = [Z^2_{1-a/2} * P * (1-P)] * Deff / d^2$$

where p is the prevalence of DM and hypertension of the epidemiology survey with 7.6% and 27.8%, respectively in Guizhou in 2010[19], *Deff* = 2, *d* = 25% × P, and the type I error rate (α) = 0.05.

The formula results in a sample size for DM of 1,495 and 409 for hypertension. With the consideration of a 10% rate of non-response, 1661 people will be recruited for DM screening and 454 for hypertension screening.

4.5 Variables of the study

Dependent and independent variables: Confirmed HTN and/or DM

Potential confounder variables: Different non-communicable diseases may be confounder to each other.

Method(s) to minimize bias(es) during study: Inclusion criteria and exclusion criteria will be performed strictly to control selection of subjects. During fieldwork investigation, induced question will be avoided to obtain intentional answer. Obscure answer is required to clarify through phone call, Internet or other methods.

4.6 Study procedure

Initially, newly diagnosed PTB cases aged 15 years or more and notified to the National Tuberculosis Program from the study site will be consecutively retrieved according to the inclusion criteria.

During the eligible patients monthly visiting the hospital to obtain their medications, TB medical staff will contact the patients to obtain informed consent and make an appointment with them for home visits.1-3 household contacts aged 15 years or more per household in home visits. Simple random sampling method will be used to select the contacts when there will be more than three adult contacts per household. Meanwhile, all the enumerated contacts will be surveyed if there were three or fewer contacts.

DM and/or hypertension will be screened by assessing systolic/diastolic (SBP/DBP) blood pressure and fasting plasma glucose/random plasma glucose (FPG/RPG) among all participants following the world health organization (WHO) standard criteria. Those with different diseases identified from the study will be transferred to the local hospitals to undergoing the related treatment based on their physical situation. Those with known diseases who have previously lapsed from treatment will be suggested to access their local hospital to continue medical services.

4.7 Statistical analysis

Data obtained from the questionnaires and medical record review will be entered into EpiData software and R software will be employed for the statistical analysis. Student's t-test or ANOVA will be used to compare age, FPG/RPG and SBP/DBP among groups as appropriate and summarized using the mean and standard deviation. For continuous variables when data are not normally distributed, the nonparametric Mann-Whitney U test will be employed. Chi-square or Fisher exact tests will be applied for categorical variates where appropriate. The univariate analysis and the multivariate logistic regression model to determine the associated factors, and will be shown through forest plots[20]. The yield was calculated by dividing number of newly detected disease by the number of subject screened excluding those known to be diseased. NNS was computed as described somewhere[21],[22].

4.8 Ethical consideration

The proposal will be approved by both the Institutional Ethics Committee of Faculty of Medicine, Prince of Songkla University, Hat Yai, Thailand, as well as the Ethics Committee of Guizhou Provincial Center for Disease Prevention and Control before the study is conducted. All proposed patients or citizens will have the right to agree or disagree to participate in the study and present using the informed consent. The anonymity and confidentiality will be maintained stringently throughout the study.

4.9 Limitation(s) and barrier(s) of the study (If applicable)

To date, no limitations and barriers are found.

5. Principal investigators

Name	Position	E-mail address
Shengqiong Guo	Investigator	179536921@qq.com
Virasakdi Chongsuvivatwong	Supervisor&<br>Investigator	cvirasak@medicine.psu.ac.th.

6. Time schedule of the study

Research activities	2019	2020

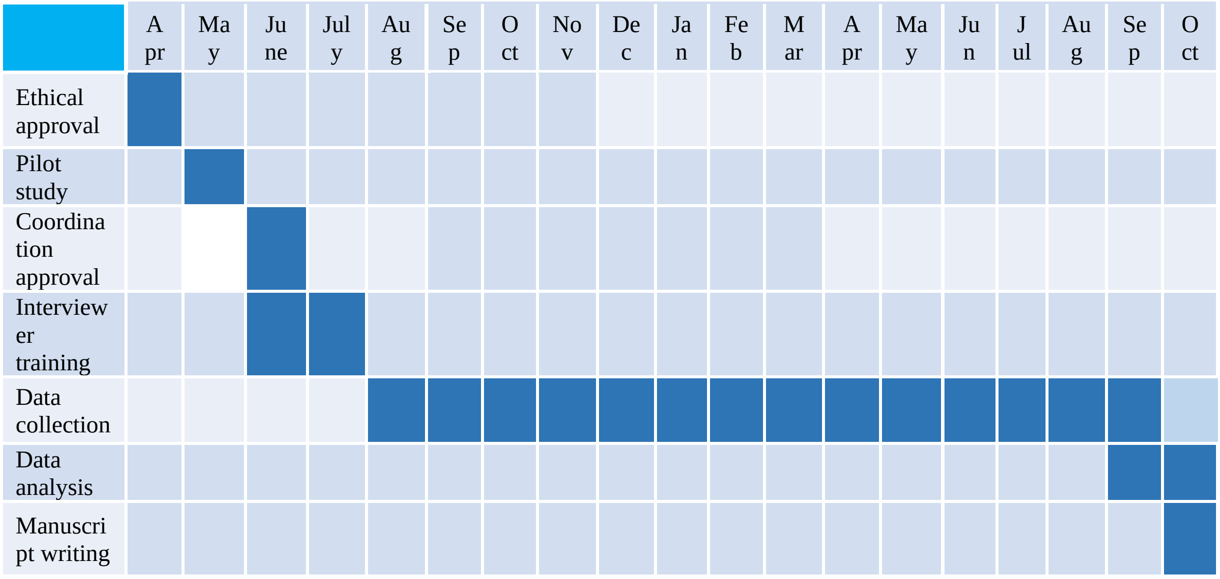

7. Budget detail of the study

 Funded by: No

Budget amount: 0(USD)

☒ Expecting funded from: Institution

Budget amount: 0(USD)

☒Private fund

Budget amount: 4692 (Baht)

**Budget category**	**Unit cost in Ren Min Bi(CNY)**	**Multiplying factors**	**Total cost(CNY)**
**1. Personnel**			
Interdepartmental collaboration	500	2*500	1000
Investigators for data collection	100	2 persons*120 days*100	24000
Participants (Gift/money)	15	(116*2) persons*15	3480
Stationary and printing service	0.5(per page for copying), 3 for one paper file per person	116*2*2	464
**Personnel Total**			**28944**

**2. Travel cost**			
Home visit	200	2 persons *2 times*200	800
Health centers visit	200	2 persons *2 times*200	800
Accommodation	300	2 persons *2 times*300	1200
**Travel Total**			**2800**
**Total budget**			
**Grand Total (CNY)**			**31744**
**Exchange rate: 1 CNY = 0.1478 (USD)**			**4692(USD)**

8. References

1. Marais BJ, Lönnroth K, Lawn SD, Migliori GB, Mwaba P, Glaziou P, et al. Tuberculosis comorbidity with communicable and non-communicable diseases: integrating health services and control efforts. The Lancet Infectious Diseases 2013;13:436–48.

2. Lim SS, Vos T, Flaxman AD, Danaei G, Shibuya K, Adair-Rohani H, et al. A comparative risk assessment of burden of disease and injury attributable to 67 risk factors and risk factor clusters in 21 regions, 1990–2010: a systematic analysis for the Global Burden of Disease Study 2010. Lancet 2012;380:2224–60.

3. Marak B, Kaur P, Rao SR, Selvaraju S. Non-communicable disease comorbidities and risk factors among tuberculosis patients, Meghalaya, India. Indian Journal of Tuberculosis 2016;63:123–5.

4. Peltzer K. Tuberculosis non-communicable disease comorbidity and multimorbidity in public primary care patients in South Africa. Afr j prim health care fam med [Internet] 2018;10. Available from: https://phcfm.org/index.php/phcfm/article/view/1651

5. Wang Q, Ma A, Han X, Zhao S, Cai J, Ma Y, et al. Prevalence of Type 2 Diabetes among Newly Detected Pulmonary Tuberculosis Patients in China: A Community

Based Cohort Study. PLoS One [Internet] 2013;8. Available from: https://www.ncbi.nlm.nih.gov/pmc/articles/PMC3867381/

6. Sharma B, Khanal VK, Jha N, Pyakurel P, Gurung GN. Study of the magnitude of diabetes and its associated risk factors among the tuberculosis patients of Morang, Eastern Nepal. BMC Public Health [Internet] 2019;19. Available from: http://www.ncbi.nlm.nih.gov/pmc/articles/PMC6873766/

7. Oni T, Youngblood E, Boulle A, McGrath N, Wilkinson RJ, Levitt NS. Patterns of HIV, TB, and non-communicable disease multi-morbidity in peri-urban South Africa- a cross sectional study. BMC Infectious Diseases [Internet] 2015;15. Available from: http://bmcinfectdis.biomedcentral.com/articles/10.1186/s12879-015-0750-1

8. Reis-Santos B, Gomes T, Macedo LR, Horta BL, Riley LW, Maciel EL. Prevalence and patterns of multimorbidity among tuberculosis patients in Brazil: a cross-sectional study. Int J Equity Health [Internet] 2013;12:61. Available from: https://www.ncbi.nlm.nih.gov/pmc/articles/PMC3765118/

9. Segafredo G, Kapur A, Robbiati C, Joseph N. Integrating TB and non-communicable diseases services: Pilot experience of screening for diabetes and hypertension in patients with Tuberculosis in Luanda, Angola. :11.

10. Ogbera AO, Kapur A, Chinenye S, Fasanmade O, Uloko A, Odeyemi K. Undiagnosed diabetes mellitus in tuberculosis: A Lagos report. Indian J Endocrinol Metab [Internet] 2014;18:475–9. Available from: https://www.ncbi.nlm.nih.gov/pmc/articles/PMC4138900/

11. Wilson J, Jungner G. The Principles and Practice of Screening for Disease. World Health Organization Public Health Papers #34 1967;22.

12. Rembold C. Number needed to screen: Development of a statistic for disease screening. BMJ (Clinical research ed) 1998;317:307–12.

13. Byrne AL, Marais BJ, Mitnick CD, Garden FL, Lecca L, Contreras C, et al. Feasibility and yield of screening for non-communicable diseases among treated tuberculosis patients in Peru. The International Journal of Tuberculosis and Lung Disease [Internet] 2018;22:86–92. Available from: http://www.ingentaconnect.com/content/10.5588/ijtld.17.0381

14. Kumpatla S, Aravindalochanan V, Rajan R, Viswanathan V, Kapur A. Evaluation of performance of A1c and FPG tests for screening newly diagnosed diabetes defined by an OGTT among tuberculosis patients—A study from India. Diabetes Research and Clinical Practice [Internet] 2013;102:60–4. Available from: https://linkinghub.elsevier.com/retrieve/pii/S0168822713002969

15. A household-level score to predict the risk of tuberculosis among contacts of patients with tuberculosis: a derivation and external validation prospective cohort study | Elsevier Enhanced Reader [Internet]. Available from: https://reader.elsevier.com/reader/sd/pii/S1473309919304232?token=19E6DF49F051464470A1ABBCC12AEE61ED3843C72DB34CD42AAC3FE6765D522C5E514F7BE521955EB653E72A82E8739E

16. Aamir AH, Ul-Haq Z, Mahar SA, Qureshi FM, Ahmad I, Jawa A, et al. Diabetes Prevalence Survey of Pakistan (DPS-PAK): prevalence of type 2 diabetes mellitus and prediabetes using HbA1c: a population-based survey from Pakistan. BMJ Open [Internet] 2019;9:e025300. Available from: https://bmjopen.bmj.com/lookup/doi/10.1136/bmjopen-2018-025300

17. Basit A, Fawwad A, Qureshi H, Shera AS. Prevalence of diabetes, pre-diabetes and associated risk factors: second National Diabetes Survey of Pakistan (NDSP), 2016–2017. BMJ Open [Internet] 2018;8:e020961. Available from: https://bmjopen.bmj.com/lookup/doi/10.1136/bmjopen-2017-020961

18. Organization WH. World Health Organization vaccination coverage cluster surveys: reference manual. World Health Organization; 2018.

19. Tao Liu, Dingming Wang, Liangxian Sun. Status of Chronic Disease and Associated Factors in Guizhou. Guizhou science and technology press, Guiiyang, 2015. https://www.amazon.cn/dp/B06XCS8BZS. https://www.amazon.cn/dp/B06XCS8BZS

20. Codewar. Codewar. How draw forestplot for regression in R. CSDN: https://blog.csdn.net/tm_ggplot2/article/details/113705379 [Internet]. Available from: https://blog.csdn.net/tm_ggplot2/article/details/113705379?ops_request_misc=%257B%2522request%255Fid%2522%253A%2522162753384816780366575335%2522%252C%2522scm%2522%253A%252220140713.130102334.pc%255Fblog.%2522%257D&request_id=162753384816780366575335&biz_id=0&utm_medium=distribute.pc_search_result.none-task-blog-2~blog~first_rank_v2~rank_v29-1-113705379.pc_v2_rank_blog_default&utm_term=%E6%A3%AE%E6%9E%97&spm=1018.2226.3001.4450

21. Kaasenbrood L, Poulter NR, Sever PS, Colhoun HM, Livingstone SJ, Boekholdt SM, et al. Development and Validation of a Model to Predict Absolute Vascular Risk Reduction by Moderate-Intensity Statin Therapy in Individual Patients With Type 2 Diabetes Mellitus. Circulation: Cardiovascular Quality and Outcomes [Internet] 2016;9:213–21. Available from: https://www.ahajournals.org/doi/full/10.1161/CIRCOUTCOMES.115.001980

22. Ho C, Breslin M, Doust J, Reid C, Nelson M. Effectiveness of blood pressure-lowering drug treatment by levels of absolute risk: Post hoc analysis of the Australian National Blood Pressure Study. BMJ Open 2018;8:e017723.

Appendix 1

Structured questionnaire for study on yield, NNS and prevalence of screening for DM and hypertension

QID______________

Start Time [ ] [ ]/ [ ] [ ]
Start Date (dd/mm/yy) [ ] [ ]/ [ ] [ ]/ [ ] [ ] [ ] [ ]

Section 1 Background's Information		
**No.**	**Information**	**Code**
A1	Index case ID	id [ ] [ ] [ ]
A2	Household ID	hid [ ] [ ] [ ]
A3	City	cit [ ] [ ]
A4	County /district	cou [ ] [ ] [ ]
A5	Village	vil [ ] [ ] [ ]
A6	Postal code	post [ ] [ ] [ ]

Section 2 Background's characteristics			
**No.**	**Question**	**Answer**	**Code**
B1	Subject type	1.Index case<br>2.household contact	subjtype [ ]
B2	Gender	1. Male 2. Female	gen [ ]
B3	Age<br>(completed year)	___________year	age [ ][ ]
B4	Ethnic<br>(Single response)	1. Han 2. Biyi<br>3. Liz 4. Tujia<br>5. Miao 6. Gelao<br>7. Yi 8. Man<br>9. Hui 10. Yao<br>11. Other (specify)_____________	eth [ ]
B5	Religion<br>(Single response)	1. No<br>2. Buddhism<br>3. Muslim<br>4. Christian<br>5. Other (Specify)_____________	reg [ ]
B6	Education level<br>(Passed level)	1. None<br>2. Read and write<br>3. Primary school<br>4. Middle school<br>5. High school<br>6. Graduate school	edu [ ]

B7	Occupation	1. Civil servant<br>2. Factory worker<br>3. Casual employee<br>4. Shopkeeper/Retail<br>5. Dependent<br>6. Student<br>7. Other_________________	occ [ ]
B8	Marital status	1. Single 2. Marriage<br>3. Divorce 4. Separate<br>5. Widowhood<br>6. Cohabitation<br>7. Other(specify)______________	mars [ ]
B9	Monthly income	_______________________CNY	inc [ ][ ][ ]
B13	Is there any history of DM in your family?	0. No (Jump to B15) 1. Yes	dmfam [ ]
B14	If yes, who has DM in your family?	1. Father<br>2. Mother<br>3. Sibling<br>4. Spouse<br>5. Grandpa<br>6. Grandma<br>7. Other (specify)______________	dmfat [ ]<br>dmmot [ ]<br>dmsib [ ]<br>dmspo [ ]<br>dmgpa [ ]<br>dmgma [ ]<br>dmoth [ ]
B15	Is there any history of HTN in your family?	0. No (Jump to B17) 1. Yes	htfam [ ]
B16	If yes, who has HTN in your family?	1. Father<br>2. Mother<br>3. Sibling<br>4. Spouse<br>5. Grandpa<br>6. Grandma<br>7. Other (specify)_____________	htfat [ ]<br>htmot [ ]<br>htsib [ ]<br>htspo [ ]<br>htgpa [ ]<br>htgma [ ]<br>htoth [ ]
B13	Is there any history of DM in your family?	0. No (Jump to B15) 1. Yes	dmfam [ ]
B14	If yes, who has DM in your family?	1. Father<br>2. Mother<br>3. Sibling<br>4. Spouse<br>5. Grandpa<br>6. Grandma<br>7. Other (specify)______________	dmfat [ ]<br>dmmot [ ]<br>dmsib [ ]<br>dmspo [ ]<br>dmgpa [ ]<br>dmgma [ ]<br>dmoth [ ]
B15	Is there any history of HTN in your family?	0. No (Jump to B17) 1. Yes	htfam [ ]
B16	If yes, who has HTN in your family?	1. Father<br>2. Mother<br>3. Sibling<br>4. Spouse<br>5. Grandpa<br>6. Grandma<br>7. Other (specify)_____________	htfat [ ]<br>htmot [ ]<br>htsib [ ]<br>htspo [ ]<br>htgpa [ ]<br>htgma [ ]<br>htoth [ ]
B17	Is there any history of COPD in your family?	0. No (Jump to B19) 1. Yes	htfam [ ]
B18	If yes, who has COPD in your family?	1. Father	htfat [ ]

		2. Mother<br>3. Sibling<br>4. Spouse<br>5. Grandpa<br>6. Grandma<br>7. Other (specify)______________	htmot [ ]<br>htsib [ ]<br>htspo [ ]<br>htgpa [ ]<br>htgma [ ]<br>htoth [ ]
B19	Is there any history of DLP in your family?	0. No (Jump to B21) 1. Yes	dlpfam [ ]
B20	If yes, who has DLP in your family?	1. Father<br>2. Mother<br>3. Sibling<br>4. Spouse<br>5. Grandpa<br>6. Grandma<br>7. Other (specify)_______________	dlpfat [ ]<br>dlpmot [ ]<br>dlpsib [ ]<br>dlpspo [ ]<br>dlpgpa [ ]<br>dlpgma [ ]<br>dlpoth [ ]
B21	Is there any history of HD in your family?	0. No (Jump to B23) 1. Yes	hdfam [ ]
B22	If yes, who has HD in your family?	1. Father<br>2. Mother<br>3. Sibling<br>4. Spouse<br>5. Grandpa<br>6. Grandma<br>7. Other (specify)_______________	hdfat [ ]<br>hdmot [ ]<br>hdsib [ ]<br>hdspo [ ]<br>hdgpa [ ]<br>hdgma [ ]<br>hdoth [ ]
B23	Is there any history of bronchitis in your family?	0. No (Jump to B25) 1. Yes	brofam [ ]
B24	If yes, who has bronchitis in your family?	1. Father<br>2. Mother<br>3. Sibling<br>4. Spouse<br>5. Grandpa<br>6. Grandma<br>7. Other (specify)______________	brofat [ ]<br>bromot [ ]<br>brosib [ ]<br>brospo [ ]<br>brogpa [ ]<br>brogma [ ]<br>brooth [ ]
B25	Is there any history of asthma in your family?	0. No (Jump to B27) 1. Yes	asthfam [ ]
B26	If yes, who has asthma in your family?	1. Father<br>2. Mother<br>3. Sibling<br>4. Spouse<br>5. Grandpa<br>6. Grandma<br>7. Other (specify)______________	asthfat [ ]<br>asthmot [ ]<br>asthsib [ ]<br>asthspo [ ]<br>asthgpa [ ]<br>asthgma [ ]<br>asthoth [ ]
B27	Is there any history of Cancer in your family?	0. No (Jump to B29) 1. Yes	cafam [ ]
B28	If yes, who has Cancer in your family?	1. Father<br>2. Mother<br>3. Sibling<br>4. Spouse<br>5. Grandpa<br>6. Grandma<br>7. Other (specify)______________	cafat [ ]<br>camot [ ]<br>casib [ ]<br>caspo [ ]<br>cagpa [ ]<br>cagma [ ]<br>caoth [ ]
B29	Is there any history of CRD in your family?	0. No (Jump to B31) 1. Yes	htfam [ ]
B30	If yes, who has CRD in your family?	1. Father	htfat [ ]

		2. Mother<br>3. Sibling<br>4. Spouse<br>5. Grandpa<br>6. Grandma<br>7. Other (specify)_____________	htmot [ ]<br>htsib [ ]<br>htspo [ ]<br>htgpa [ ]<br>htgma [ ]<br>htocrd [ ]

Section 3 Health related characteristics

No.	Question	Answer	Code
C1	Smoking	1. Current smoker<br>2. Smoked in the past (Jump to C4)<br>3. Never smoker (Jump to C5)	smok [ ]
C2	If smoker, number of cigarettes per day	__________________cigarettes	ncig [ ]
C3	Duration of smoking	__________________years	dura [ ]
C4	If ex-smoker, when stopped smoking?	__________________years	exsmo [ ]
C5	Passive smoker	1. No<br>2. Very few times in week<br>3. Few times in week<br>4. Very few times daily<br>5. Few times daily<br>6. Most of times daily	passmo [ ]
C6	Drinking	1. Current drinker<br>2. Never drinker (Jump to C9)<br>3. Drunk in the past (Jump to C10)	drin [ ]
C7	Duration of drinking	__________________years	drindura [ ]
C8	If current drinker, frequency of drinking during 1 year	1. Daily<br>2. 5-6 days / week<br>3. 3-4 days / week<br>4. 1-2 days / week<br>5. 2-3 times/ month<br>6. Once a month	drinfreq [ ]
C9	If ex-drinker, when stopped drinking?	__________________years	exdrin [ ][ ]
C10	Do you know how many grams of salt an adult can eat at most per day?	1.Yes<br>0. No (Jump to C12)	saltkn [ ]
C11	If yes, how many grams of salt an adult can eat at most per day?	1. 5g<br>2. 6g (If not correct, inform the value to the participant)<br>3. 7g 4. 8g 5. 9g	saltgr [ ]
C12	Do you know how many grams of edible oil an adult can eat at most per day?	1.Yes<br>0. No (Jump to C14)	oilkn [ ]
C13	If yes, how many grams of edible oil an adult can eat at most per day?	1. 25-30g (If not correct, inform the value to the participant)<br>2. 30-35g 3. 35-40g<br>4. 40-45g 5. 45-50g	oilgr [ ]
C14	How often do you eat fruits / vegetables?	1. Daily<br>2. 5-6 days / week<br>3. 3-4 days / week<br>4. 1-2 days / week	fruitfreq [ ]

		5. 2-3 times/ month<br>6. Once a month<br>7. Never	
C15	Do you often do physical exercise?	1.Yes<br>0. No (Jump to C17)	exerci [ ]
C16	If yes, how often do you do physical exercise?	1. Daily<br>2. 5-6 days / week<br>3. 3-4 days / week<br>4. 1-2 days / week<br>5. 2-3 times/ month<br>6. Once a month	exercifreq [ ]
C17	Do you have anything that depresses or pleases you?	0. No 1.Yes	anythi [ ]
C18	Do you often stay up late?	1.Yes<br>0. No (Jump C20)	stayup [ ]
C19	If yes, how often do you stay up late?	1. Daily<br>2. 5-6 days / week<br>3. 3-4 days / week<br>4. 1-2 days / week<br>5. 2-3 times/ month<br>6. Once a month	stayupfreq [ ]
C20	What do you think about your health status?	1. Very good 2. Good<br>3. So so 4. Bad<br>5. Very bad	healsta [ ]

Section 4 History relevant to NCDs

No.	Question	Answer	Code
D1	Weight loss	0. No 1.Yes	weiloss [ ]
D2	Loss appetite	0. No 1.Yes	appede [ ]
D3	Increasing appetite	0. No 1.Yes	appein [ ]
D4	Intake more water	0. No 1.Yes	watem [ ]
D5	Eat more food	0. No 1.Yes	foodm [ ]
D6	Urinate more	0. No 1.Yes	urinm [ ]
D7	Repeated infection	0. No 1.Yes	urinm [ ]
D8	Limb ulceration	0. No 1.Yes	urinm [ ]
D9	Renal function impairment	0. No 1.Yes	urinm [ ]
D10	Blurred vision	0. No 1.Yes	urinm [ ]
D11	Headache	0. No 1.Yes	headac [ ]
D12	Dizziness	0. No 1.Yes	dissy [ ]
D13	irritability	0. No 1.Yes	irrita [ ]

D14	palpitations	0. No 1.Yes	palpi [ ]
D15	Nausea	0. No 1.Yes	nause [ ]
D16	Vomiting	0. No 1.Yes	vomi [ ]
D17	Chronic cough	0. No 1.Yes	coug [ ]
D18	Expectoration	0. No 1.Yes	expe [ ]
D19	Chest tightness	0. No 1.Yes	chestt [ ]
D20	Breathlessness	0. No 1.Yes	breat [ ]
D21	Dyspnea	0. No 1.Yes	dysp [ ]
D22	Have you ever been diagnosed as HTN?	0. No (Jump to D28)<br>1.Yes	htndia [ ]
D23	If you have ever been diagnosed as HTN, how long?	_________________year	htntim [ ]
D24	If yes, have you ever controlled HTN?	0. No 1.Yes	htncont [ ]
D25	Where were you diagnosed as HTN?	1. Government hospital<br>2. Registered private clinic<br>3. Traditional healer<br>4. Other (specify)______________	htnplace [ ]
D26	Have you ever been diagnosed as DM?	0. No (Jump to D32)<br>1.Yes	dmdia [ ]
D27	If you have been diagnosed as DM, how long?	______________years only	dmtim [ ]
D28	If yes, have you ever controlled DM?	0. No 1.Yes	dmcont [ ]
D29	Where were you diagnosed as DM?	1. Government hospital<br>2. Registered private clinic<br>3. Traditional healer<br>4. Other (specify)______________	dmplace [ ]
D30	Have you ever been diagnosed as COPD?	0. No (Jump to D36)<br>1.Yes	copddia [ ]
D31	If you have ever been diagnosed as COPD, how long?	_________________year	copdtim [ ]
D32	If yes, have you ever controlled COPD?	0. No 1.Yes	copdcont [ ]
D33	Where were you diagnosed as COPD?	1. Government hospital<br>2. Registered private clinic<br>3. Traditional healer<br>4. Other (specify)______________	copdplace [ ]
D34	Have you ever been diagnosed as DLP?	0. No (Jump to D40) 1.Yes	dlpdia [ ]
D35	If you have been diagnosed as other DLPs, how long?	______________years only	dlptim [ ]
D36	If yes, have you ever controlled DLP?	0. No 1.Yes	dlpcont [ ]
D37	Where were you diagnosed as DLP?	1. Government hospital<br>2. Registered private clinic<br>3. Traditional healer<br>4. Other (specify)______________	dlplace [ ]
D38	Have you ever been diagnosed as HD?	0. No (Jump to D44)<br>1.Yes	hddia [ ]

D39	If you have been diagnosed as other HD, how long?	_____________years only	hdtim [ ]
D40	If yes, have you ever controlled HD?	0. No 1.Yes	hdcont [ ]
D41	Where were you diagnosed as HD?	1. Government hospital<br>2. Registered private clinic<br>3. Traditional healer<br>4. Other (specify)_____________	hdplace [ ]
D42	Have you ever been diagnosed as cancer?	0. No (Jump to D48)<br>1.Yes	cadia [ ]
D43	If you have been diagnosed as other cancer, how long?	_____________years only	catim [ ]
D44	If yes, have you ever controlled cancer?	0. No 1.Yes	cacont [ ]
D45	Where were you diagnosed as cancer?	1. Government hospital<br>2. Registered private clinic<br>3. Traditional healer<br>4. Other (specify)_____________	dlplace [ ]
D46	Have you ever been diagnosed as CRD?	0. No (Jump to E1) 1.Yes	crddia [ ]
D47	If you have been diagnosed as CRD, how long?	_____________years only	crdtim [ ]
D48	If yes, have you ever controlled CRD?	0. No 1.Yes	crdcont [ ]
D49	Where were you diagnosed as CRD?	1. Government hospital<br>2. Registered private clinic<br>3. Traditional healer<br>4. Other (specify)_____________	crdplace [ ]
**Extra Characteristics for Household Contacts**			
E1	Did you share a room with your TB family member in the past 3 months?	0. No 1.Yes	sharoo [ ]
E2	Did you care your TB family member in the past 3 months?	0. No 1.Yes	carefa [ ]
**The end**			

Thank you for your participation.

End time [ ] [ ]/ [ ] [ ] End Date (dd/mm/yy) [ ] [ ]/ [ ] [ ]/ [ ] [ ] [ ] [ ]

Signature of investigator_________________________
